# Dietary Bioactive Compounds and Human Health and Disease

**DOI:** 10.3390/nu12020348

**Published:** 2020-01-29

**Authors:** Sonia González

**Affiliations:** 1 Department of Functional Biology, University of Oviedo, 32762 Oviedo, Spain; soniagsolares@uniovi.es; Tel.: +34-985-104-209; 2 Diet, Microbiota and Health Group, Instituto de Investigación Sanitaria del Principado de Asturias (ISPA), 32762 Oviedo, Spain

In the last century, solid scientific evidence has demonstrated the role of nutritional compounds in the maintenance of health. However, in the last few decades, the interest in the nutritional field has gone a step forward, searching for novel compounds with the capacity to reduce the risk of non-communicable diseases. In this context, the special issue of nutrients entitled “*Dietary bioactive compounds and human health and disease*” compiles 4 revisions and 12 original articles reporting the most novel findings of the impact of different bioactive compounds on a wide range of pathologies as different as non-alcoholic fatty liver disease, cancer, and obesity. This issue covers a hot topic, which is evidenced by the large number of recent articles compiled in the reviews.

There is no consensus in the literature to define the term “bioactive compound”, although within the most widely accepted denominations they are “compounds which have the capability and the ability to interact with one or more component(s) of living tissue by presenting a wide range of probable effects” [1]. Despite that this concept has been highly dynamic over time, this collection of review articles provides a useful summary, especially of the progress in the roles of phenolic compounds on health.

Regarding polyphenols, revisions of different kinds have been compiled. Den Hartogh et al. summarizes in vitro and in vivo the most relevant studies examining the nephroprotective effects of resveratrol. For this purpose, the authors list the major articles studying the impact of resveratrol administration on each of the cell types present in the nephron, depending on the concentration administered and the duration of the intervention [2]. Mayo et al. review the potential role of microorganisms as mediators in the biosynthesis of equol, one of the main metabolites of isoflavones, discussing their possible application to various pathologies and proposing possible modes of action for these compounds [3]. This is a very interesting work that shows that, regardless of the daizein consumed with the diet, the amount of bioactive compounds generated from it has a high inter-individual variation, which may be conditioned by the human intestinal microbiota. Finally, Dabeek et al. extensively review the existing literature on the bioavailability and bioactivity of dietary quercetin and kaempferol, two of the most consumed polyphenols [4,5], assessing their possible impact on cardiovascular health in humans [4]. Together with phenolics, sulphur-containing compounds have also accumulated a large body of scientific evidence of their protective role against pathologies related to oxidative stress, inflammation, and the immune system, many of which are summarized by Rodrigues et al. [6].

At the metabolic level, in animal models, Lim et al. find a decrease in insulin resistance, triglycerides and LDL and total cholesterol, white fat tissue weights, and leptin levels after supplementation with cyanidine-3-O-galactoside-enriched *Aronia melanocarpa* extract [7]. Although these results are not yet extrapolated to humans, the high content in phenolic compounds of this berry and its antioxidant capacity, in comparison to other fruits [8], makes it interesting in the study of oxidative stress-related pathologies. In the same line, Zhu et al. propose konjac mannan oligosaccharides as an alternative dietary intervention for the delay of obesity, diabetes, and their associated complications [9] and Mun et al. propose the *Curcuma Longa* water extract as a potential agent against non-alcoholic fatty liver disease through modulating fatty acid uptake [10].

In addition to these vegetable compounds, this issue includes novel findings regarding the implication of bioactive peptides, amino acids, and fatty acids in different metabolic outcomes. Mas-Capdevila et al. raise interesting hypotheses about how the resistance of peptides to gastric digestion may modulate their antihypertensive effect [11], adding a novel approach to previous work about this topic [12,13]. At this point, factors such as bioavailability, synergistic effects between these compounds, or their interaction with other food components, are essential to deepening the knowledge of bioactive compounds on health [4]. In this regard, Mc Veay et al. evaluate the synergic effect of lauric acid and L-tryptophan on fasting glucose, insulin, and glucagon in healthy men [14].

Among the most innovative works, the findings of Lim Y et al. report the beneficial effect at a vascular level and blood pressure in chronic smokers after supplementation with Sanghuang–Danshen bioactive compounds [15], and Kuban-Jankowska et al. identify docosahexaenoic (DHA) acid as a potential agent in breast cancer prevention [16].

If some of the preliminary findings compiled in this special issue are confirmed in the future, they could be of great interest to the agro-food industry. To this end, there is a need to identify optimal extraction methods. This is the case with Altieri et al., who carry out a pilot study to obtain high value-added products for the industry by means of the transformation of by-products of pomegranate juice through a hydro-alcoholic extraction [17]. The obtaining of algae or plant-derivate products with potential effects on different diseases such as infections in the upper respiratory tract, [18] or even in non-communicable diseases such as cancer [16] or non-alcoholic fatty liver disease [10], could be a promising way to obtain new agents to fight these pathologies.
